# Lysine Acetylome Profiling Reveals Diverse Functions of Acetylation in Deinococcus radiodurans

**DOI:** 10.1128/spectrum.01016-21

**Published:** 2022-08-16

**Authors:** Yongqian Zhang, Nuomin Li, Qiushi Wei, Rui Min, Feng Liu, Fuli Wang, Yulin Deng

**Affiliations:** a School of Life Science, Beijing Institute of Technology, Beijing, People’s Republic of China; b State Key Laboratory of NBC Protection for Civilian, Beijing, People’s Republic of China; c Beijing Key Laboratory for Separation and Analysis in Biomedicine and Pharmaceuticals, Beijing Institute of Technology, Beijing, People’s Republic of China; University of Guelph

**Keywords:** *Deinococcus radiodurans*, lysine acetylation, DNA damage, posttranslational modification

## Abstract

Lysine acetylation is a highly conserved posttranslational modification that plays essential roles in multiple biological functions in a variety of organisms. Deinococcus radiodurans (D. radiodurans) is famous for its extreme resistance to radiation. However, few studies have focused on the lysine acetylation in D. radiodurans. In the present study, antibody enrichment technology and high-resolution liquid chromatography mass spectrometry are used to perform a global analysis of lysine acetylation of D. radiodurans. We create the largest acetylome data set in D. radiodurans to date, totally identifying 4,364 lysine acetylation sites on 1,410 acetylated proteins. Strikingly, of the 3,085 proteins annotated by the uniport database, 45.7% of proteins are acetylated in D. radiodurans. In particular, the glutamate (G) preferentially appears at the −1 and +1 positions of acetylated lysine residues by motif analysis. The acetylated proteins are involved in metabolic pathways, propanoate metabolism, carbon metabolism, fatty acid metabolism, and the tricarboxylic acid cycle. Protein-protein interaction networks demonstrate that four clusters are involved in DNA damage repair, including homologous recombination, mismatch repair, nucleotide excision repair, and base excision repair, which suggests that acetylation plays an indispensable role in the extraordinary capacity to survive high levels of ionizing radiation. Taken together, we report the most comprehensive lysine acetylation in D. radiodurans for the first time, which is of great significance to reveal its robust resistance to radiation.

**IMPORTANCE** D. radiodurans is distinguished by the most radioresistant organism identified to date. Lysine acetylation is a highly conserved posttranslational modification that plays an essential role in the regulation of many cellular processes and may contribute to its extraordinary radioresistance. We integrate acetyl-lysine enrichment strategy, high-resolution mass spectrometry, and bioinformatics to profile the lysine acetylated proteins for the first time. It is striking that almost half of the total annotated proteins are identified as acetylated forms, which is the largest acetylome data set reported in D. radiodurans to date. The acetylated proteins are involved in metabolic pathways, propanoate metabolism, carbon metabolism, fatty acid metabolism, and the tricarboxylic acid cycle. The results of this study reinforce the notion that acetylation plays critical regulatory roles in diverse aspects of the cellular process, especially in DNA damage repair and metabolism. It provides insight into the roles of lysine acetylation in the robust resistance to radiation.

## INTRODUCTION

Protein posttranslational modifications (PTMs) are a major regulatory mechanism in the cell (1). PTMs increase the functional diversity of the proteome and change the protein characteristics, such as enzymatic activity, subcellular localization, stability, degradation, and protein-protein interaction. The main posttranslational modifications currently discovered in prokaryotes and eukaryotes are phosphorylation, acetylation, succinylation, glycosylation, and ubiquitination and so on, which almost affect all aspects of cell activities from genotype to functional phenotype. The protein acetylation was first discovered in 1963, and it can occur at the alpha amino group of the N-terminal protein or the ε-amino moiety of lysyl side chains of protein by enzymatic and nonenzymatic mechanism (2). Lysine acetylation and deacetylation are controlled by two groups of enzymes, lysine acetyltransferases (KATs) and lysine deacetylases (KDACs) (3). In enzymatically catalyzed Lys acetylation reactions, KATs catalyze the transfer of an acetyl group from acetyl coenzyme A (CoA) to the ε-amino group of protein lysine residues, whereas the KDACs activity removes the acetyl group from the modified lysine residue.

Over the past decade, the new approaches to identify the acetylated proteins and their acetylated sites in the proteome have been developed based on the liquid chromatography-tandem mass spectrometry. In particular, the development of pan-acetyl-lysine antibody successfully realizes the enrichment of acetylated peptides, thereby overcoming technical challenges caused by the low abundance of acetylated peptides in the cell. So far, acetylome have been extensively analyzed and identified in a wide range of organisms, such as Escherichia coli (4–7), *Thermus thermophiles* (8), C. albicans (9), Bacillus subtilis (10), *Drosophila* (11), Staphylococcus aureus (12), Bombyx mori (13), *Cyanobacterium* (14), *Thrips* (15), *Rice* (16), and Homo sapiens (3), which indicate that lysine acetylation is a highly evolutionarily conserved PTMs from prokaryotes to eukaryotes. These studies also showed that acetylation plays a vital role in regulating gene expression, DNA repair, protein translation, and energy metabolism (17).

Deinococcus radiodurans is famous for its extraordinary resistance to ionizing radiation and oxidative stress. It can survive at a radiation dose of 5 kGy with little loss of viability (18). The strong resistance of radiation is a combination of multiple mechanisms in D. radiodurans, which may have fast and effective DNA repair capabilities (19–21), efficient reactive oxygen species (ROS) clearance (22–24), and various protein protection mechanisms (redox balance) (25–28). PTMs may be involved in regulating these biological processes and pathways. It has been reported that the succinylome analysis has been investigated and 492 succinylation sites in 270 proteins are identified in D. radiodurans (29). However, little is known about the global lysine acetylation in D. radiodurans, which is of great significance to reveal its robust resistance to radiation. In the present study, we integrated acetyl-lysine enrichment strategy, high-resolution mass spectrometry, and bioinformatics to profile the lysine-acetylated proteins at the proteome level.

## RESULTS

### Proteome analysis of acetylated proteins and sites in D. radiodurans.

**FIG 1:**
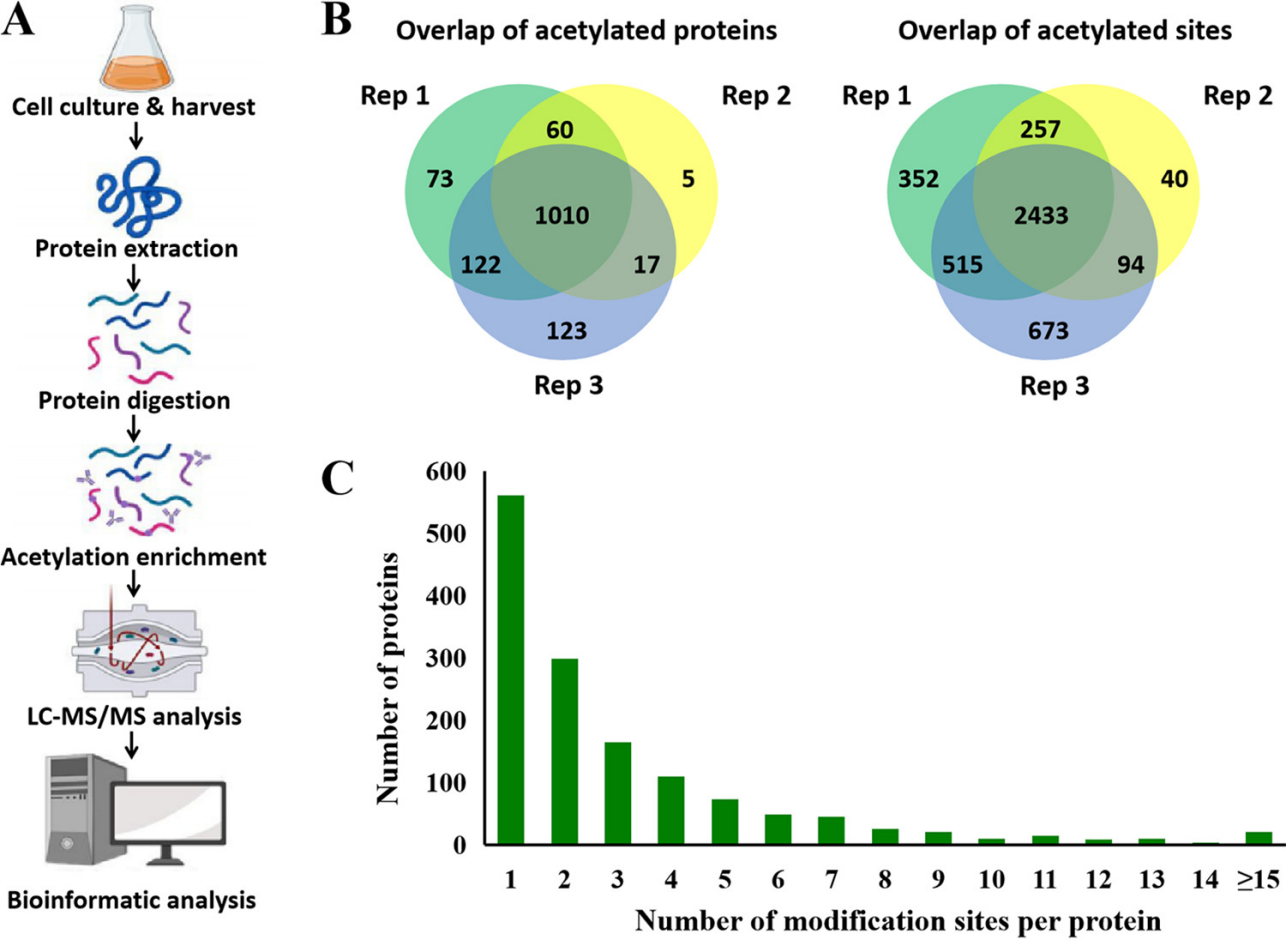
Proteome-wide identification of lysine acetylation in D. radiodurans. (A) The experimental flowchart for comprehensive analysis of lysine acetylome in D. radiodurans. The cartoon is created with BioRender.com. (B) Overlap of acetylated proteins and acetylated sites in three biological replicates. (C) Distribution of lysine acetylated proteins with respect to the number of sites of acetylation in a protein.

To systematically determine the protein acetylation sites in D. radiodurans, an integrated approach involving trypsin digestion, anti-acetyl antibody based affinity enrichment and high-resolution liquid chromatography-tandem mass spectrometry (LC-MS/MS) analysis was performed (Fig. 1A). The scheme is depicted in Fig. 1A. To assess the depth and reproducibility of acetylome in D. radiodurans, we performed the proteomics analyses by three biological replicates (Fig. 1B). In total, 4,364 acetylation sites on 1,410 acetylated proteins were identified (Table S1). Theoretically, there are 3,085 predicted proteins encoded by D. radiodurans genome. It was surprising that 45.7% of the total predicted proteins were acetylated. The number of lysine acetylation sites in proteins was diverse and distributed from 1 to 15 (or more than 15) in Fig. 1C. The results indicated 561 proteins with only 1 acetylation site, which accounted for 40% of acetylated proteins. The percentages of proteins with two, three, and four or more modification sites were 21%, 11%, and 28%, respectively.

### Cellular localization of acetylated proteins and analysis of acetylation sites.

**FIG 2:**
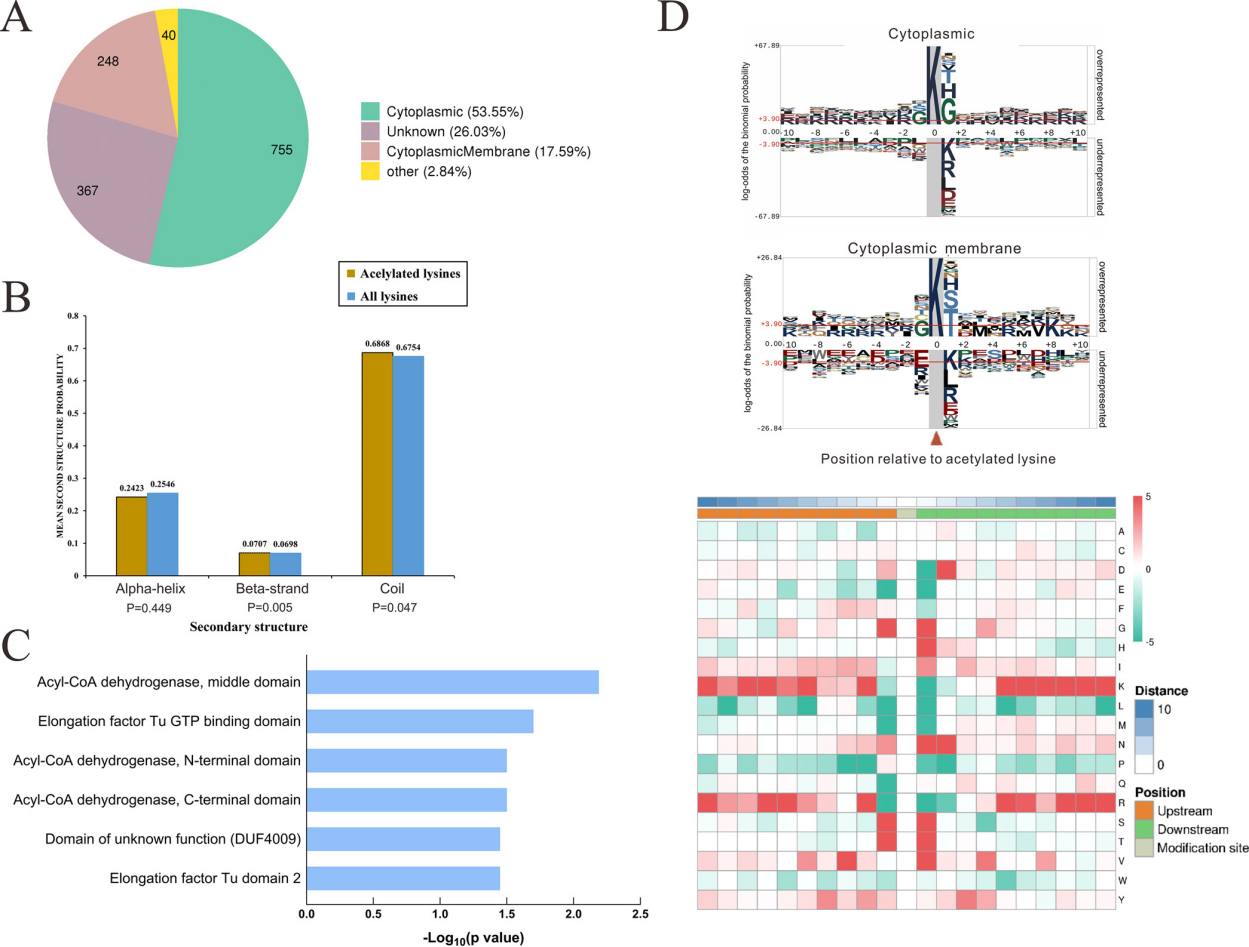
The characteristics of acetylated proteins and sites. (A) subcellular localization of the acetylated protein. (B) Probabilities of lysine acetylation in different protein secondary structures (alpha-helix, beta-strand, and coil). (C) Protein domain enrichment of proteins corresponding to acetylation sites. (D) The motif enrichment heatmap of upstream and downstream amino acids of all identified acetylated sites.

To gain further insights into the lysine acetylome in D. radiodurans, we performed subcellular localization using CELLO software (Table S2). As shown in Fig. 2A, most acetylated proteins were located in the cytoplasm (53.55%) and cytoplasmic membrane (17.59%), respectively. Only 2.84% of proteins were assigned as other locations (2.84%). However, there were still 26.03% proteins unknown about their subcellular localization. These results implied that the acetylated proteins have diverse functions in D. radiodurans. Then, we further probed the local secondary structures of lysine acetylation. As shown in Fig. 2B, 24.2% of acetylated sites were located in α-helix and 7.1% in β-strand. In addition, the majority of acetylation sites were located on coils (68.7%), which was similar to previous studies in Bacillus amyloliquefaciens (30) and Thermus thermophilus (8).

Moreover, the domain of acetylated proteins was enriched using Pfam analysis to determine the functional enrichment. As shown in Fig. 2C, six domain families were enriched for acetylated lysine and were predominantly composed of the middle domain of acyl-CoA dehydrogenase and GTP binding domain of elongation factor Tu, indicating that lysine acetylation is involved in cell metabolism and protein translation. To explore the acetylation motifs in D. radiodurans, the context of amino acids surrounding the acetylated lysine was investigated in our data set. We found that 3,817 peptides included the amino acid sequence from the −10 to the +10 positions surrounding the acetylated lysine. We also found that the frequency of glycine was preferentially in positions −1 and +1 in cytoplasm. Furthermore, the motifs K^ac^S, K^ac^T, and K^ac^H (where K^ac^ represents the acetylated lysine) were located in the downstream of acetylated lysine. In the cytoplasmic membrane, the motifs K^ac^S, K^ac^T, and K^ac^H ranked at the top three in the downstream, while glycine was still dominant in the upstream. Consistent with our observations, the occurrence of glycine and serine in positions −1 or +1 was highest in Sulfolobus islandicus (31) and *Nonion thrips* (15), confirming that lysine acetylation is a highly conserved modification in different species. The heat map of amino acid compositions surrounding the acetylation sites is depicted in Fig. 2D. Red indicates that this amino acid is significantly enriched near the acetylated site, and green indicates that this amino acid is significantly reduced near the acetylated site. It was concluded that proteins with G, S, and T but without K, R, L, M, and E surrounding lysine residues will be preferentially modified by lysine acetyltransferase in D. radiodurans.

**FIG 3:**
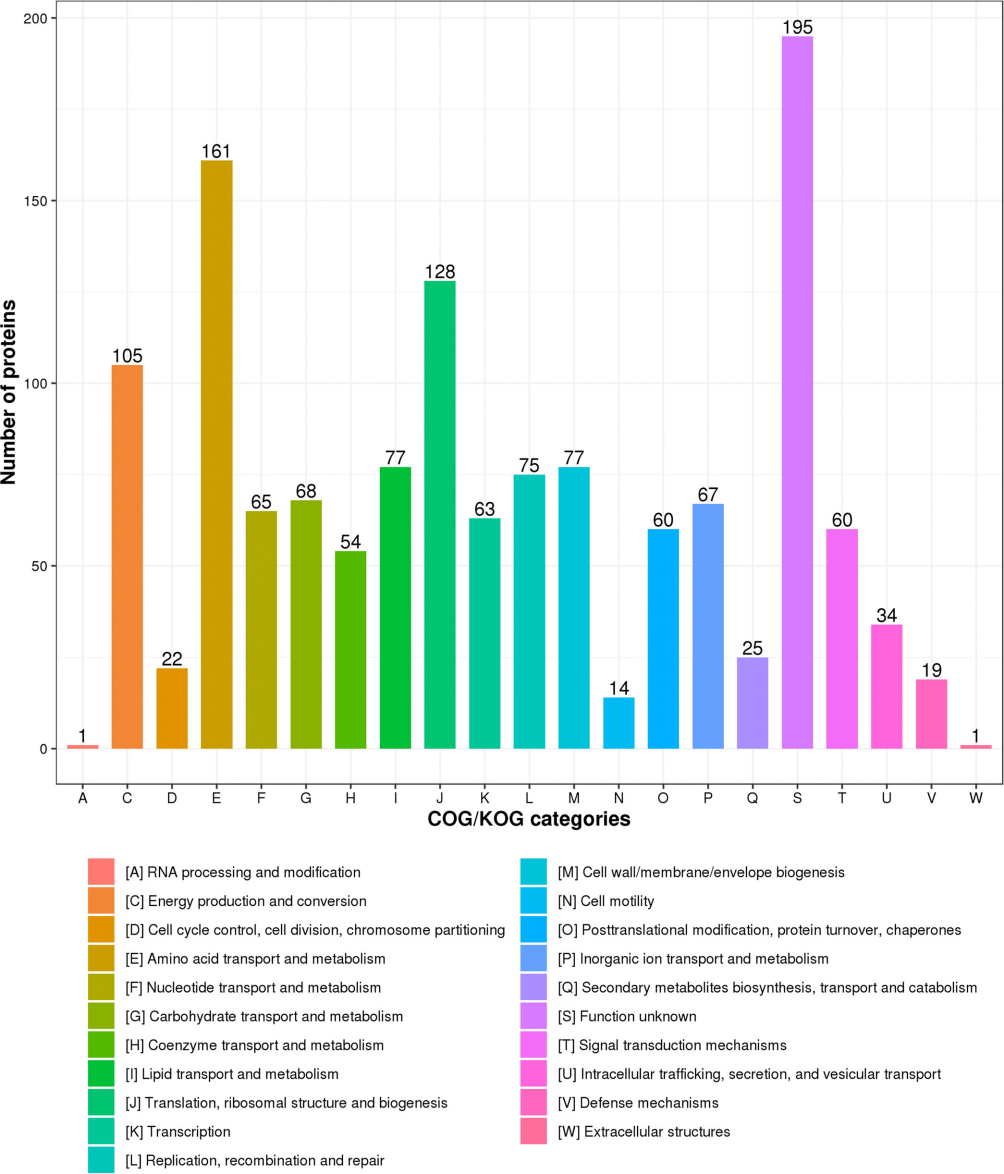
COG functional classification of acetylated proteins.

Since the functions of the majority of proteins are unknown in D. radiodurans, we further explored the Clusters of Orthologous Groups of proteins (COG) function of acetylated proteins. In our case, 1,410 acetylated proteins were annotated by Egg-NOG analysis (Table S3). As shown in Fig. 3, the top three categorizations of acetylated proteins were involved in the amino acid transport and metabolism [E]; translation, ribosome structure, and biogenesis [J]; and energy production and conversion [C].

**FIG 4:**
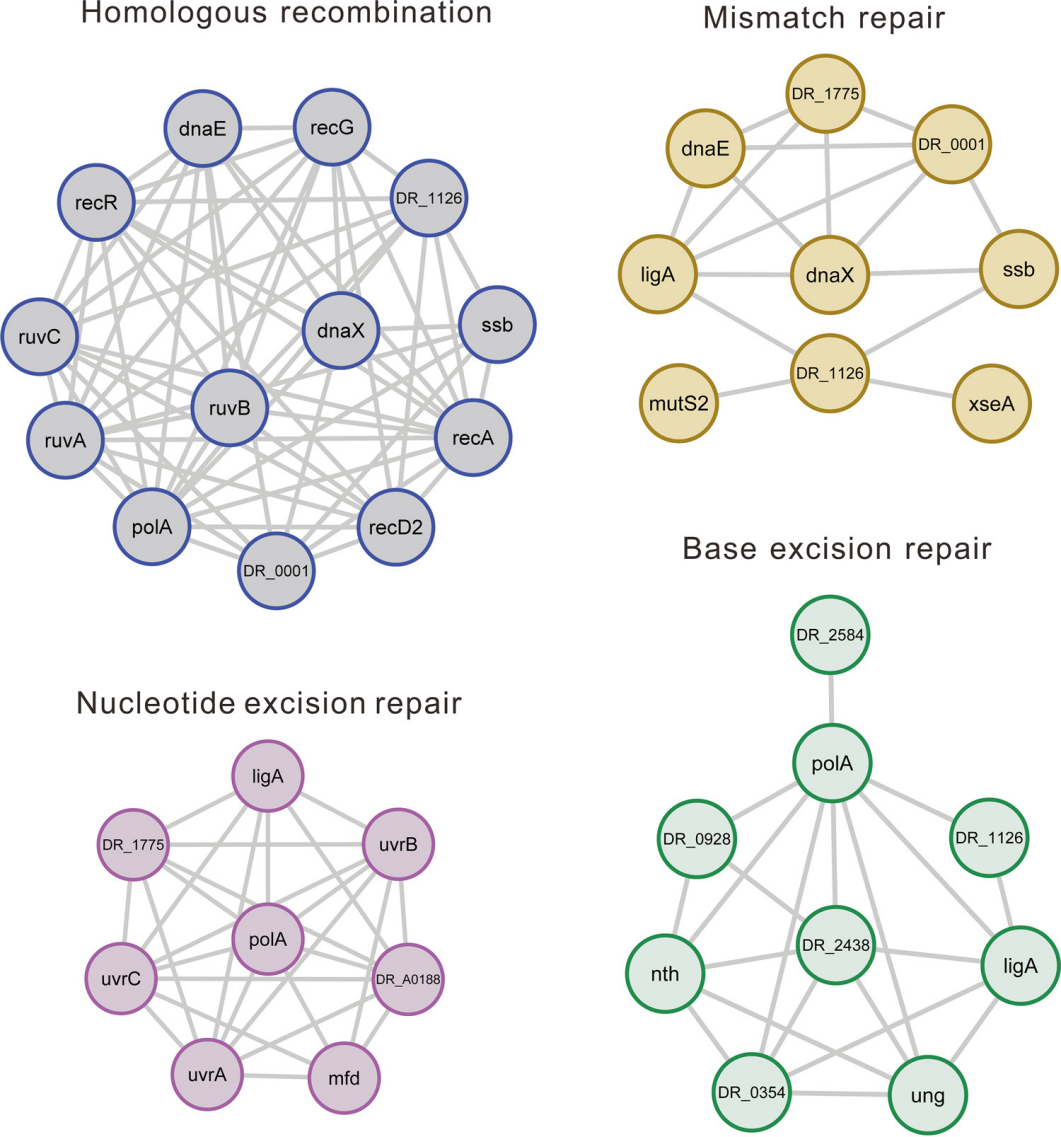
The networks generated by 28 acetylated proteins are significantly enriched in homologous recombination, mismatch repair, nucleotide excision repair, and base excision repair.

As D. radiodurans has fast and effective DNA repair ability, 28 acetylated proteins in the replication, recombination, and repair category [L] attracted us (Table S4). Protein-protein interaction networks were retrieved from STRING database to obtain the functional connections and elucidate the potential radio-resistant mechanism in D. radiodurans. The four clusters involved in DNA damage repair were obtained, including homologous recombination, mismatch repair, nucleotide excision repair, and base excision repair, which suggested that acetylation played an indispensable role in regulating the DNA damage repair (Fig. 4).

### Functional annotation of identified acetylated proteins.

**FIG 5:**
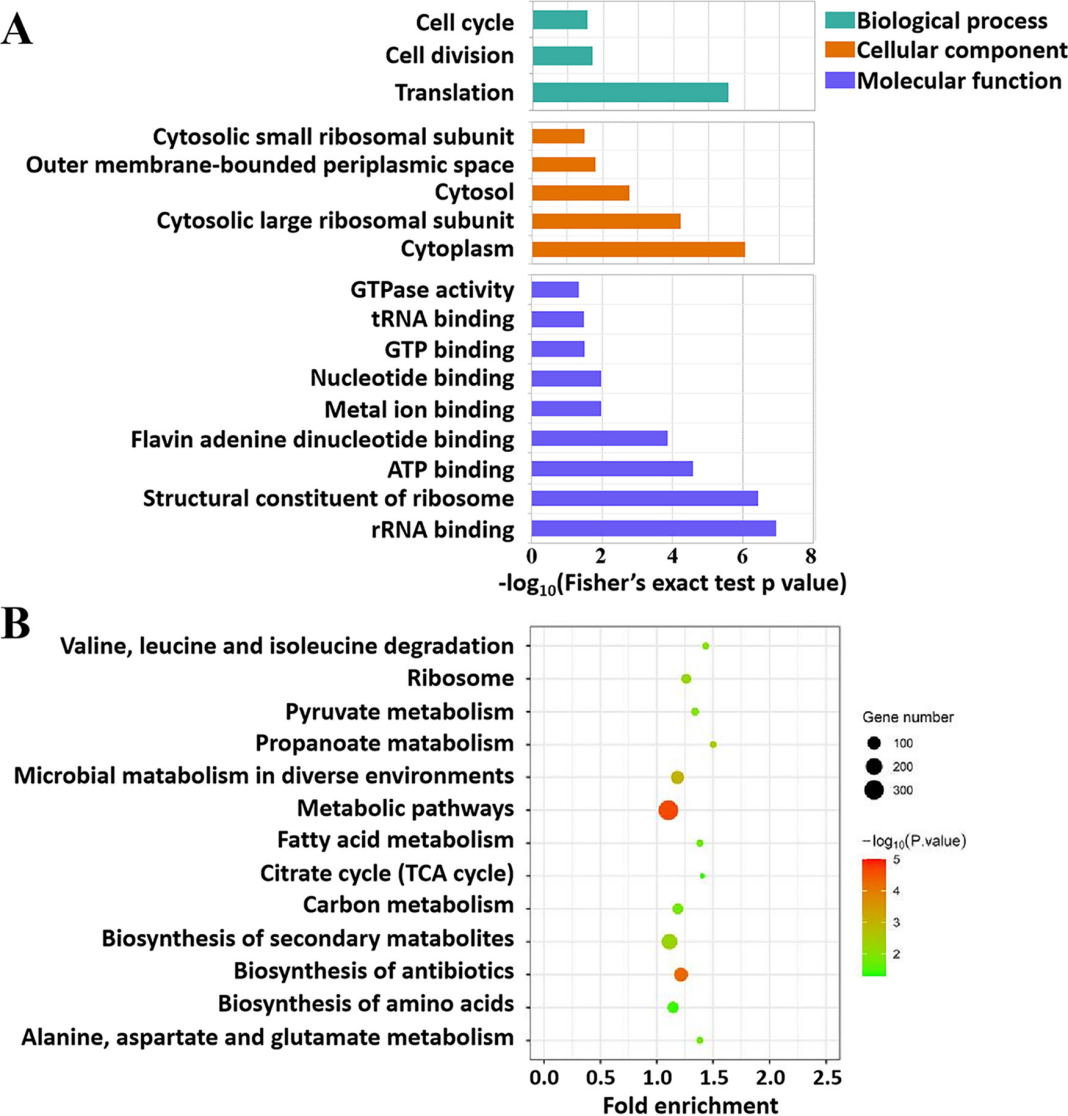
Functional analysis of acetylated proteins in D. radiodurans. (A) The classification of acetylated proteins based on biological process, cellular component, molecular function. The column length represents the DAVID fold enrichment score. (B) The enriched KEGG pathways are shown along with *P* value and gene number. The color represents the significance of enrichment.

To better understand the possible roles of the acetylation in global, acetylated proteins in D. radiodurans were assigned to biological process, cellular component, and molecular function (Fig. 5A). In the biological process classification, the top three enriched categories of acetylated proteins were translation (*P* value = 2.78E-06), cell cycle (*P* value = 0.02), and cell division (*P* value = 0.03). A large number of acetylated proteins were found to be related to the cytoplasm (*P* value = 9.57E-07) and cytosolic large ribosomal subunit (*P* value = 6.12E-05) based on cellular component. The molecular function analysis revealed that the largest proportion of acetylated proteins was related to rRNA binding (*P* value = 1.18E-07), followed by structural constituent binding of the ribosome (*P* value = 3.75E-07) and ATP binding (*P* value = 2.58E-05).

As shown in Fig. 5B, Kyoto Encyclopedia of Genes and Genomes (KEGG) pathway analysis revealed that the top categorizes of acetylated proteins were enriched in the metabolic pathways (316 acetylated proteins, *P* values = 2.32E-05). Most of the enzymes involved in the metabolic pathways of fatty acid synthesis/degradation, TCA cycle, glycolysis/gluconeogenesis, and pyruvate metabolism were found to be acetylated (Fig. S1), which indicated that acetylation plays an essential role in every aspect of cell metabolism in D. radiodurans. For example, many enzymes involved in the metabolism of fatty acids were found to be acetylated, including propionyl-CoA carboxylase alpha subunit (10 acetylated sites) and propionyl-CoA carboxylase beta subunit (8 acetylated sites). Strikingly, each enzyme in every step of TCA cycle has acetylated sites. For example, citrate synthase, aconitate hydratase A, isocitrate dehydrogenase, and pyruvate dehydrogenase have 10 or more acetylation sites. Furthermore, acetylated proteins were also involved in the biosynthesis of antibiotics and amino acids. Taken together, the results of Gene Ontology (GO) functional analysis and KEGG pathway indicate that the lysine acetylation has diverse protein functions and biological categories in D. radiodurans.

### Protein-protein interaction network of the identified acetylated proteins.

**FIG 6:**
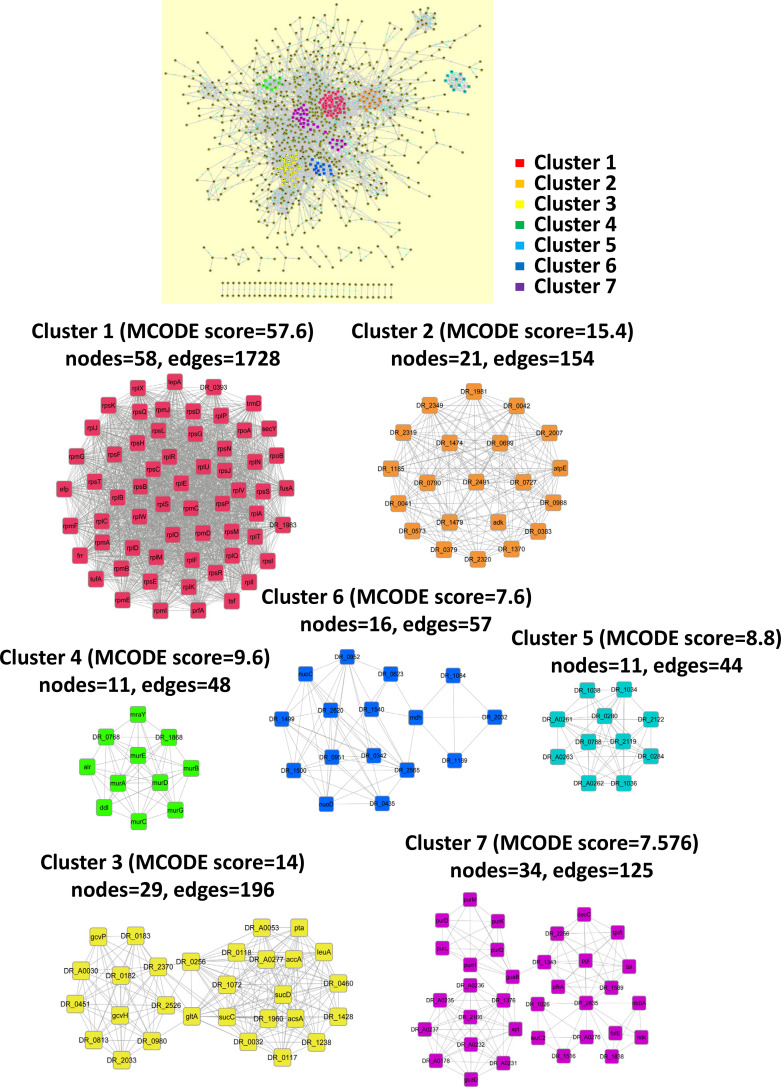
Protein-protein interaction networks for 1,410 acetylated proteins in D. radiodurans, which are constructed using the Cluepedia plugin and STRING database with STRING score threshold of 0.7. The networks generated by the acetylated proteins are significantly enriched in seven clusters, including pyruvate metabolism, purine metabolism, propanoate metabolism, carbon metabolism, ribosome, ABC transporters and peptidoglycan biosynthesis.

To further understand the protein-protein interaction network, we used the Search Tool for the Retrieval of Interacting Genes/Proteins (STRING) database to obtain the functional interactions based on 1,410 acetylated proteins. The global protein-protein interaction is depicted in Fig. 6. It was found that several protein complexes and functions were tightly clustered. Among these clusters, seven clusters were highlighted, including pyruvate metabolism, purine metabolism, propanoate metabolism, carbon metabolism, ribosome, ABC transporters, and peptidoglycan biosynthesis.

### Cross talk between lysine acetylation and succinylation in D. radiodurans.

**FIG 7:**
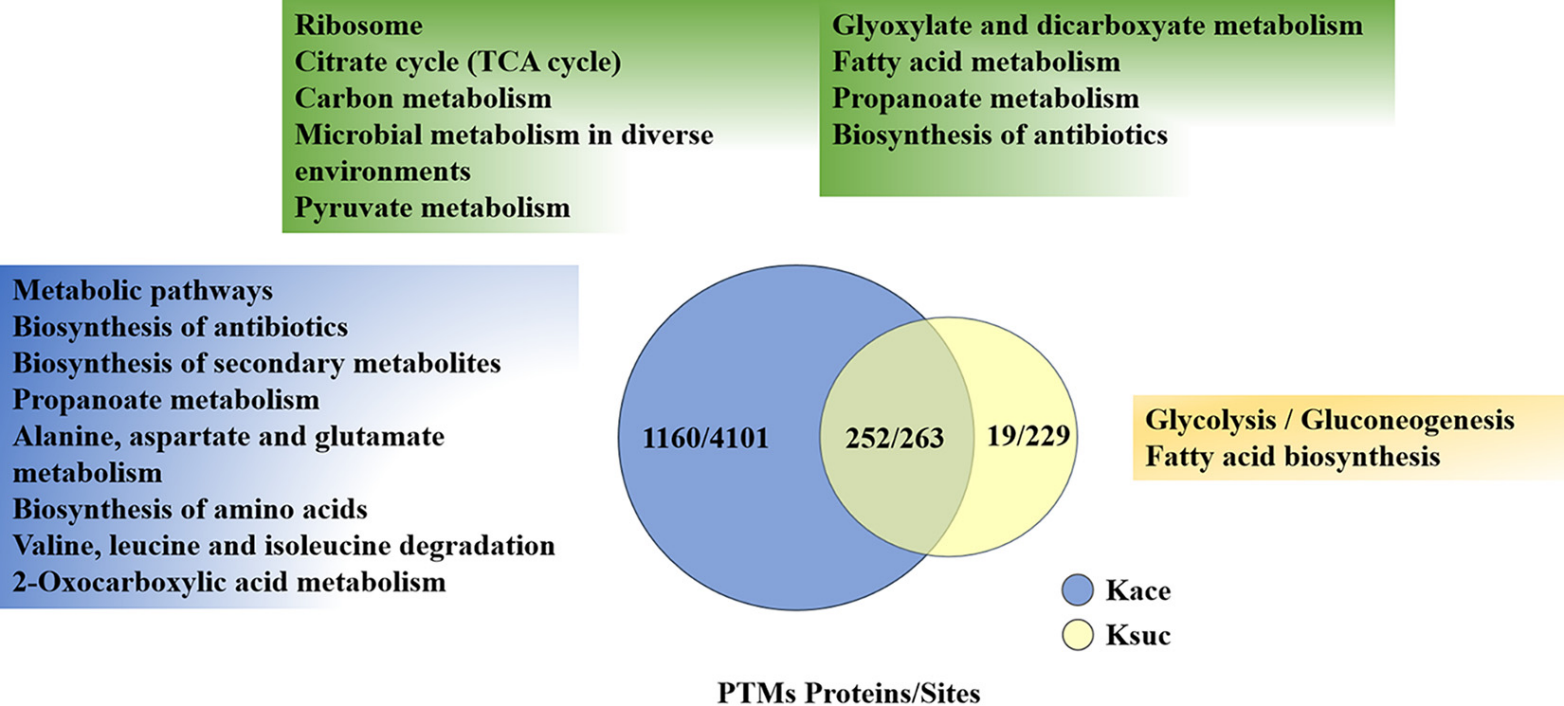
Venn diagram displays the overlap number of proteins and sites between the lysine acetylome and succinylome in D. radiodurans. The Kace and Ksuc represent lysine acetylation and succinylation, respectively. The blue and yellow areas show the KEGG pathways associated with 1,160 acetylated and 19 succinylated proteins, respectively. The green area shows the common KEGG pathways corresponding to 252 proteins.

Previous studies found that the lysine was coregulated by multiple PTMs cross talk such as acetylation and succinylation in both prokaryotes and eukaryotes. It has been found that there are 492 succinylation sites in 270 proteins in D. radiodurans. To investigate any potential cross talk between lysine acetylation and succinylation, we identified 263 sites on 250 proteins that were overlapped in D. radiodurans (Fig. 7 and Table S5).

The findings implied that the acetylation and succinylation occasionally occurred at the same lysine residues. Furthermore, the KEGG pathways were enriched based on their overlapped and unique modified proteins. The top three pathways of overlapping proteins were associated with ribosome, TCA cycle, and carbon metabolism. Several pathways were enriched by proteins unique to lysine acetylation, such as metabolic pathways, biosynthesis of antibiotics, and biosynthesis of secondary metabolites, etc., whereas glycolysis/gluconeogenesis and fatty acid biosynthesis were enriched by proteins unique to lysine succinylation. These results indicate that the physiological functions of two PTMs were coregulated in D. radiodurans.

### Lysine acetylation of proteins of D. radiodurans exposure to radiation.

To test whether the acetylation level of proteins was dynamically regulated in response to radiation, the bacteria were cultured and exposed to 6 kGy of ^60^Co gamma rays at a dose rate of 30 Gy/min (Peking University, Beijing, China). Two different strategies were utilized to quantify the acetylated proteins in D. radiodurans upon radiation.

**FIG 8:**
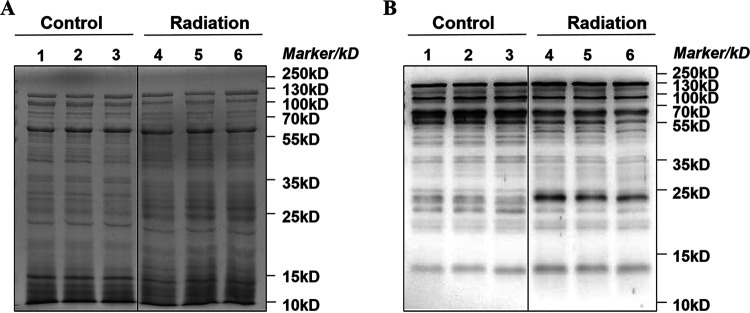
The overall proteins were stained by Coomassie both in control and postirradiation group at 4 h (A). Loading amount is 20 μg protein per lane. After run the SDS-PAGE, the gel was transferred and incubated with anti-acetyllysine antibody and corresponding second antibody (B).

First, the level of global proteins and acetylated proteins was performed by Coomassie staining and anti-Western blot, respectively. It was observed that the profile of overall proteins was not dramatically changed between the control and postirradiation group at 4 h from the Coomassie staining gel in Fig. 8A. However, as shown in Fig. 8B, the acetylation level of some proteins was obviously decreased in postirradiation group at 4 h, especially the protein bands between 55 kDa and 70 kDa. These results demonstrated that the level of protein acetylation was dynamically regulated in response to radiation.

**FIG 9:**
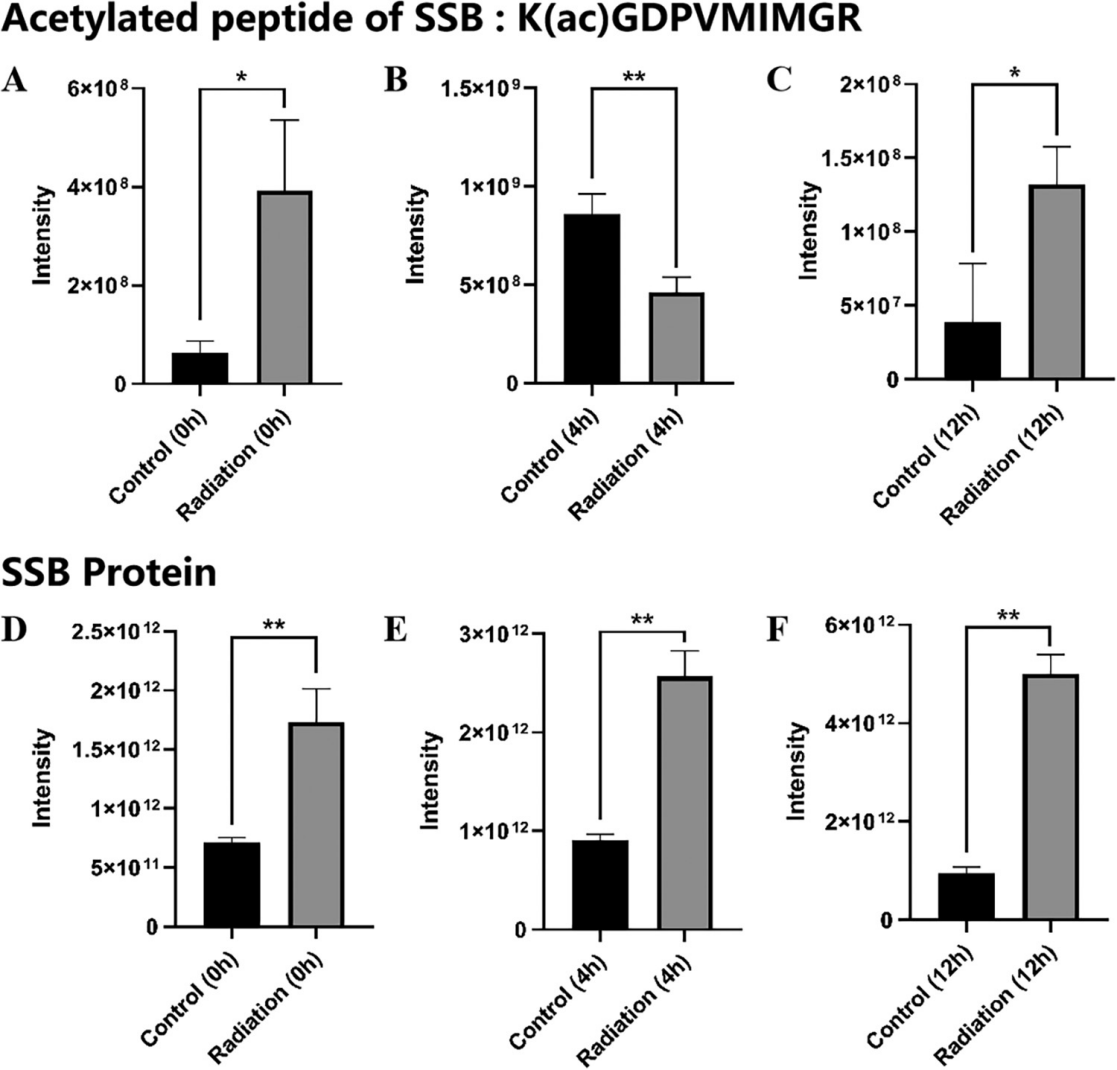
The label-free quantification of acetylated peptide of single-stranded DNA-binding protein (SSB) (A to C) and SSB protein (D to F) at 0 h, 4 h, and 12 h both in control and postirradiation group. Each group was performed on D. radiodurans in triplicate (*n* = 3). *, *P* < 0.05 or **, *P* < 0.01 versus control group.

Second, to investigate which acetylated proteins was dynamically regulated in response to radiation, the label-free quantitative proteomic strategy was performed to quantify the differentially expressed proteins between the control and postirradiation groups. Mass spectrometry-based analysis revealed that one acetylated site was identified on single-stranded DNA-binding protein (SSB). The SSB protein is essential for ionizing radiation resistance and plays an important role in DNA replication, recombination and repair. As shown in Fig. 9A to C, the amount of acetylated peptide of SSB was dynamically regulated upon the radiation. Compared with control, the amount of acetylated peptide of SSB was downregulated at 4 h but was upregulated both at 0 h and 12 h in the postirradiation group. However, the amount of SSB protein was all significantly upregulated in the postirradiation group at different time points compared with control (Fig. 9D to F). This indicates that acetylation might serve as an important posttranslational modification to regulate the function of SSB protein in response to the radiation, at least to some extent.

## DISCUSSION

D. radiodurans is famous for its extraordinary resistance to ionizing radiation and oxidative stress. The robust resistance to radiation is due to the coordination of various mechanisms such as fast and effective DNA repair capabilities, efficient ROS clearance, and a high concentration of divalent manganese ions. PTMs may be involved in regulating these biological processes and pathways. It has been reported that the succinylome analysis has been investigated and 492 succinylation sites in 270 proteins are identified in D. radiodurans (29). However, little is known about the global lysine acetylation, which is of great significance to reveal its robust resistance to radiation.

In the present study, we integrated acetyl-lysine enrichment strategy, high-resolution mass spectrometry and bioinformatics to profile the lysine acetylated proteins at the proteome level and for the first time provide the comprehensive analysis of lysine acetylation in D. radiodurans. A total of 4,364 acetylation sites on 1,410 proteins were identified. It was striking that 45.7% of the 3,085 total predicted proteins were acetylated. Our research found that acetylated proteins are mostly located in the cytoplasm and cytoplasmic membrane. According to the analysis of acetylation motifs, acetylated proteins with G, S, and T but without K, R, L, M, and E surrounding lysine residues will be preferentially modified by lysine acetyltransferase in D. radiodurans.

The KEGG and protein-protein interaction network analysis revealed that acetylation occurred on many metabolic enzymes and the key proteins involved in DNA damage repair. The acetylation could regulate the activities of enzymes in central metabolism and involve in response to different types of DNA damage repair in the postirradiation recovery phase. Thus, the acetyltransferases and deacetylases might be the possible targets for developing the novel antiradiation strategies, although the two kinds of enzyme sequences have not been reported so far in D. radiodurans.

Functional enrichment results indicate that protein acetylation involves many metabolic pathways, mainly including translation, glycolysis, and tricarboxylic acid cycles, in which the rate-limiting enzymes are mostly acetylated, and the specific functions of acetylation need to be investigated in further study (12, 30–32).

Previously, lysine acetylation has been demonstrated to be evolutionarily conserved in prokaryotes. To evaluate the conservation of acetylated proteins, we compared the acetylated proteins of *E.coli* (7) and D. radiodurans. The acetylated proteins are considered to be homologous proteins between two types of bacterial as the max bitcore is more than 35%. As shown in Fig. S2A, a total of 273 proteins were overlapped, which was considered evolutionarily conserved acetylated proteins (Table S6). To gain deeper insight into these proteins, KEGG analysis was performed by DAVID. As shown in Fig. S2B, the top three pathways were ribosome, biosynthesis of antibiotics, and RNA degradation, respectively.

We further investigated the cross talk between lysine acetylation and succinylation in D. radiodurans. Although the number of acetylated proteins that we identified in the present study is much more than that of succinylated proteins in the study of Zhou et al. (29), it is not neglected this is possibly due to the high affinity of antibody and the evolution of mass spectrometry in recent years. The KEGG analysis revealed that lysine acetylation and succinylation not only have their unique physiological function but also coregulated some key cellular metabolic pathways in D. radiodurans. The results of this study provide insight into the role of lysine acetylation in the robust resistance to radiation.

To summarize, we reported the largest acetylome data set in D. radiodurans for the first time using antibody enrichment technology and high-resolution liquid chromatography mass spectrometry. Our findings illustrate the possible functions and regulatory roles of acetylation, which is of great significance to reveal its robust resistance to radiation.

## MATERIALS AND METHODS

### Strains and growth conditions.

D. radiodurans was purchased from CGMCC (No.1.633). The strains were grown in TGY medium (1% tryptone, 0.5% glucose, 0.1% yeast extract) at 30°C and 150 rpm/min. The strains were harvested in a logarithmic period, and its optical density at 600 nm (OD_600_) is about 1. The bacterial solution was centrifuged at 10,000 × *g* at 4°C for 10 min, and the bacterial precipitation was collected. The cells were washed twice with PBS buffer and cells were collected by centrifugation.

### Protein extraction.

The collected bacteria were dissolved in lysis buffer (8 M urea, 2% complete protease inhibitor mixture, 1% deacetylase inhibitor mixture). The cells were broken by ultrasonic wave (instrument type, Sonics vcx800; power, 800 W; frequency, 20 kHz; processing time, 4 min; vibration, 2 s; interval, 2 s), the broken cell solution was centrifuged at 12,000 × *g* at 4°C for 10 min, and the supernatant was removed. The supernatant was precipitated with 20% TCA at −20°C for 2 h. The supernatant was discarded after centrifugation at 15,000 × *g* at 4°C for 10 min. The precipitate was washed with ice-cold acetone three times and redissolved in buffer (8 M urea and 50 mM NH_4_HCO_3_). The protein concentration was determined by using the BCA kit (Thermo) and then stored at −80°C.

### Trypsin digestion.

Approximately 2 mg protein was reduced with 10 mM DTT at 56°C for 30 min and subsequently alkylated with 50 mM iodoacetamide for 30 min at room temperature in dark. After diluting urea to less than 2 M with 50 mM NH_4_HCO_3_, the trypsin was added at a trypsin-to-protein mass ratio of 1:50 at 37°C overnight, and then trypsin was added at a trypsin-to-protein mass ratio of 1:100 for an additional 4 h. Next, trypsin was inactivated by addition of 1% formic acid and concentrated in a vacuum.

### The enrichment of acetylated peptides.

The peptides were redissolved in NETN buffer (100 mM NaCl, 1 mM EDTA, 50 mM Tris HCl, 0.5% Nonidet P-40 [pH 8.0]) and centrifuged at 12,000 × *g* at 4°C for 10 min, and the supernatant was collected. The peptide solution was mixed with prewashed anti-acetyl-lysine antibody beads (PTM Bio) at 4°C for 4 h with gentle shaking. The beads were centrifuged at 1,000 × *g* at 4°C for 1 min, washed four times with NETN buffer, and then washed twice with deionized water. The acetylated peptides were eluted with 1% trifluoroacetic acid, repeated three times, and combined with the eluate three times. The resulting peptides were desalted with Monospin C_18_ column (GL Sciences Inc.).

### LC-MS/MS analysis.

The peptides were analyzed by nanoelute coupled with timsTOF Pro mass spectrometer. Mobile phase A consisted of 0.1% formic acid, 98% H_2_O, and 2% ACN and mobile phase B consisted of 0.1% formic acid, 10% H_2_O, and 90% ACN. A 60-min gradient (mobile phase B: 0 to 42 min, from 7% to 24% B; 42 to ~54 min, from 24% to 32% B; 54 to ~57 mim, 32% to 80% B; 57to ~60 min, 80% B) was used at a flow rate of 450 nl/min. The data were acquired in a data-dependent mode. For mass spectrometry parameters, the *m/z* range was set to 350 to 1,800 for the MS scan, and the accumulation time was 0.25 s. The top 10 most intense ions in MS1 were selected for MS/MS analysis and the dynamic exclusion time was 24 s.

### Data analysis.

The mass spectrometry files were processed using Maxquant. The protein database of D. radiodurans is the uniport proteome_UP000002524 protein data set downloaded from UniProtKB (https://www.uniprot.org/) on 2 October 2019. The mass tolerance for precursor ions was set as 20 ppm in First search and 5 ppm in Main search, respectively. The specific cleavage enzyme was Trypsin KR_C, allowing up to 4 missing cleavages. The fixed modification was carbamidomethyl [C] and the variable modifications were oxidation [M] and acetyl [ProteinN-term] and acetyl [K]. A maximum false discovery rate of 1% was employed for the identification of a protein bioinformatics analysis.

### Bioinformatics analysis.

**(i) Subcellular localization.** The subcellular localization was predicted by CELLO. The localizations included the cytoplasmic and the cytoplasmic membrane and others. There are also some proteins with unknown localization in D. radiodurans.

**(ii) Secondary structure.** Secondary structure analysis was carried out using NetSurfP. Only predictions with the probability > 0.5 for one of the different secondary structures (alpha-helix, β-strand, and coil) were considered significant.

**(iii) Domain analysis.** Domain annotation of lysine-acetylated proteins was performed using InterProScan based on sequence similarity and the InterPro domain database (http://www.ebi.ac.uk/interpro/). Protein domains with a corrected *P* value of <0.05 were considered significant.

**(iv) Motif analysis.** Soft MoMO (motif-x algorithm) was utilized to analyze the model of sequences constituted with amino acids in specific positions of modify-21-mers (10 amino acids upstream and 10 downstream of the acetylated sites) in all protein sequences.

**(v) GO annotation.** Gene ontology regarding the biological process (GOBP), GO cellular components (GOCC), and GO molecular function (GOMF) terms were derived from the UniProt-GOA database (http://www.ebi.ac.uk/GOA/).

**(vi) KEGG pathway.** Kyoto Encyclopedia of Genes and Genomes (KEGG) database (https://www.kegg.jp/) was used to annotate the pathways in D. radiodurans. A two-sided hypergeometric test with a Benjamini-Hochberg correction was performed to assess enrichment significance. Only pathways with a *P* value <0.05 were presented.

**(vii) Protein-protein interaction network.** The protein-protein interaction (PPI) information was generated from the STRING database. We used Cytoscape software plugin ClueGO and Cluepedia to analyze the interaction network of acetylated proteins. All analyses with a corrected *P* value of <0.05 were considered significant. To reduce data redundancy, the kappa score of PPI was also set to 0.7.

### Lysine acetylation of proteins of D. radiodurans exposure to radiation.

**(i) The growth and radiation conditions.** The strains were grown in TGY medium (1% tryptone, 0.5% glucose, 0.1% yeast extract) at 30°C and 150 rpm/min. For the radiation experiment, the strains were exposed to 6 kGy of ^60^Co gamma rays at a dose rate of 30 Gy/min (Peking University, Beijing, China). Subsequently, the nonirradiated control and irradiated samples were centrifuged (10,000 × *g*, 5 min, 4°C) and transferred to fresh TGY medium at an initial OD_600_ of 0.1 for postirradiation recovery. The bacteria were collected at 0 h, 4 h, and 12 h during postirradiation recovery. The cells were washed twice with PBS buffer and harvested after centrifugation (10,000 × *g*, 10 min, 4°C). The cell pellets were snap-frozen in liquid nitrogen and stored at −80°C until further proteomic analysis.

**(ii) SDS-PAGE and Western blot.** The protein concentrations both in control and postirradiation recovery groups were analyzed through Bradford assay (Bio-Rad, USA). SDS-PAGE gel was prepared to separate proteins and the overall proteins were stained by Coomassie to guarantee the same loading amount between the control and postirradiation samples. The gel was transferred to 0.22-μm polyvinylidene fluoride (PVDF) membrane by electroblotting. After blocking in 5% skim milk for 2 h at room temperature, the membrane was incubated with anti-acetyl-lysine antibody (PTM Bio, China) at 4°C overnight. Furthermore, the blot was incubated with horseradish peroxidase-conjugated mouse IgG secondary antibody (Pierce, USA) for 1 h at room temperature after washing. Blots were developed using an ECL kit (Millipore, USA) following the manufacturer's protocol.

**(iii) High-performance liquid chromatography-electrospray ionization-tandem mass spectrometry.** The peptide mixture was dissolved in water containing 0.1% FA and analyzed using an online EASY-nL-LC 1000 coupled with an Orbitrap Q-Exactive HFX mass spectrometer (Thermo Fisher Scientific, MA, USA). Peptides were separated using a 15-cm house-made C_18_ reversed-phase column (100-μm inner diameter, 1.9 μm resin) and a 120-min elution gradient. Mobile phase A consisted of 0.1% FA, 2% ACN, and 98% H_2_O, while mobile phase B consisted of 0.1% FA, 2% H_2_O, and 98% ACN. A 90-min gradient (mobile phase B: 5% at 0 min, 10% at 16 min, 22% at 60 min, 35% at 78 min, 99% at 83 min, 99% at 86 min, 5% at 90 min) was used at a flow rate of 300 nl/min. The data were obtained in a data-dependent mode. For mass spectrometry parameters, the *m/z* range was set to 350 to 1,500 for the MS scan with accumulation time zero.25 s. The top 20 most intense peptides in MS1 were selected for MS/MS analysis. The scan range of MS/MS was set as 100 to 3,000 *m/z*, and the dynamic exclusion time was 20 s.

**(iv) Data analysis.** The RAW mass spectrometry files were processed using pfind against the Uniprot D. radiodurans database downloaded from UniProtKB (https://www.uniprot.org/) on 22 October 2019. The built-in label-free quantification algorithm in pFind was applied to quantification. The following parameters were used: the precursor and fragment tolerance were both set to 20 ppm. Enzyme specificity was set as Trypsin/P with a maximum of three missed cleavages; Oxidation (M) Acetylation (Protein N-term) and Acetylation (K) was searched as variable modification and Carbamidomethyl (C) was searched as fixed modification; the other parameters in pFind were set with default values. The differentially abundant proteins (SSB protein) and the acetylated peptides of SSB were quantified by unpaired two-tailed Student's *t* test with a significance threshold set at *P* value of <0.05 and fold change of intensities calculated as postirradiation/control group by three biological replicates.

**Data availability.** The proteomics data have been deposited to the ProteomeXchange Consortium (http://proteomecentral.proteomexchange.org) via the PRIDE partner repository with the data set identifier PXD035309.
