# Supplementary material for: Lysine Acetylome Profiling Reveals Diverse Functions of Acetylation in Deinococcus radiodurans
Source: Microbiol Spectr. 2022 Aug 16;10(5):e01016-21. doi: 10.1128/spectrum.01016-21 (PMC9603093; doi:10.1128/spectrum.01016-21)
Supplement: Supplemental file 7 — Supplemental material. Download spectrum.01016-21-s0001.pdf, PDF file, 0.4 MB [file spectrum.01016-21-s0001.pdf]

Supplemental material

Fig.S1 Lysine acetylation in every aspect of cell metabolism.

Fig.S2 Evolutionary conservation analysis of acetylated proteins between *D. radiodurans* and *E.coli*.

Table S1 Acetylated proteins and acetylated sites in three biological replicates.

Table S2 Subcellular localization of acetylated proteins.

Table S3 Acetylated proteins annotated by Egg-NOG.

Table S4 Four clusters involved in DNA damage repair.

Table S5 Lysine acetylome and succinylome in *D. radiodurans.*

Table S6 The evolutionarily conserved acetylated proteins between *D. radiodurans* and *E.coli*

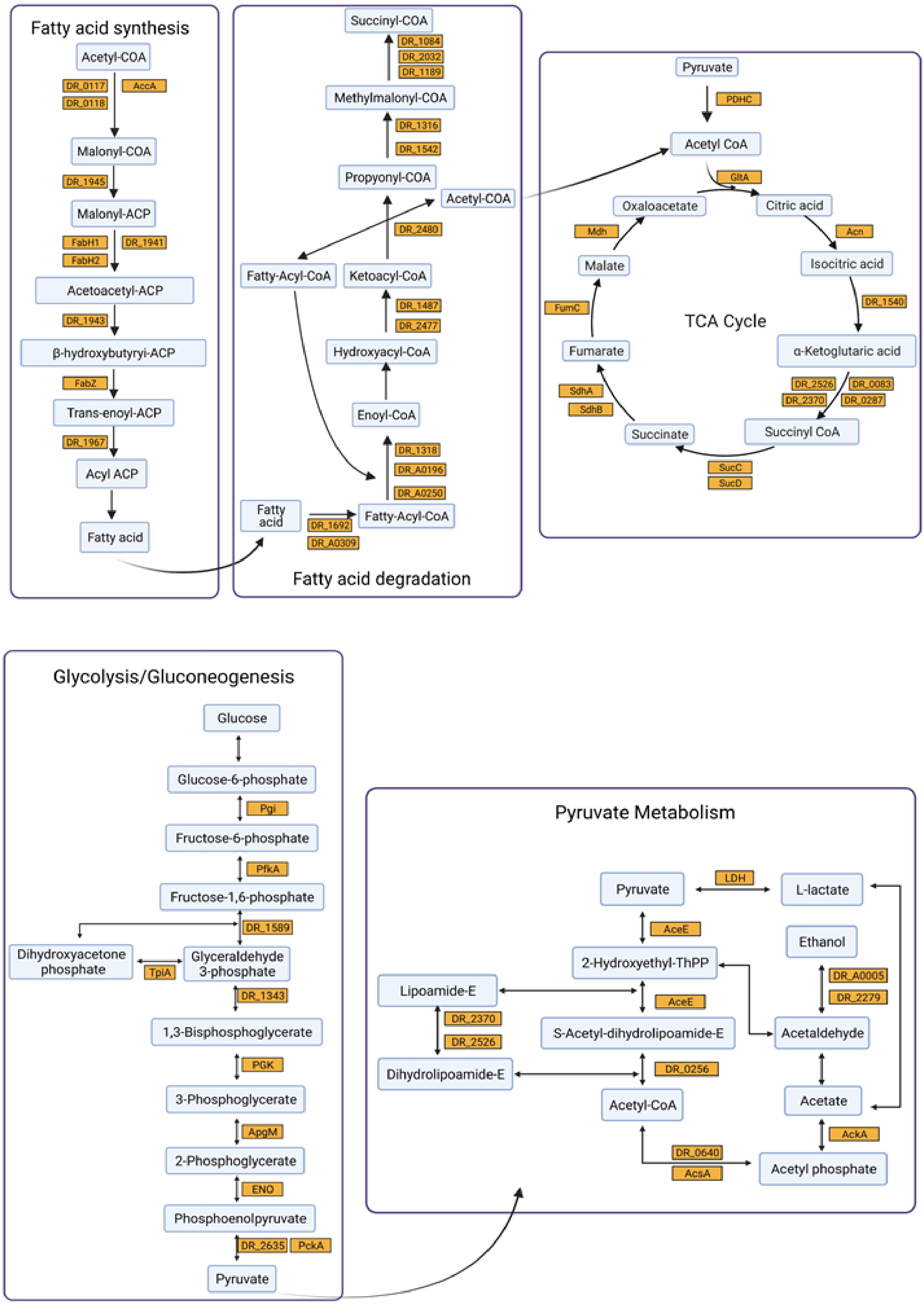

FIG.S1 Lysine acetylation plays a critical role in every aspect of cell metabolism including fatty acid synthesis/degradation, TCA cycle, glycolysis/gluconeogenesis, and pyruvate metabolism. The yellow indicates that the proteins (or enzymes) were acetylated in *D. radiodurans.*

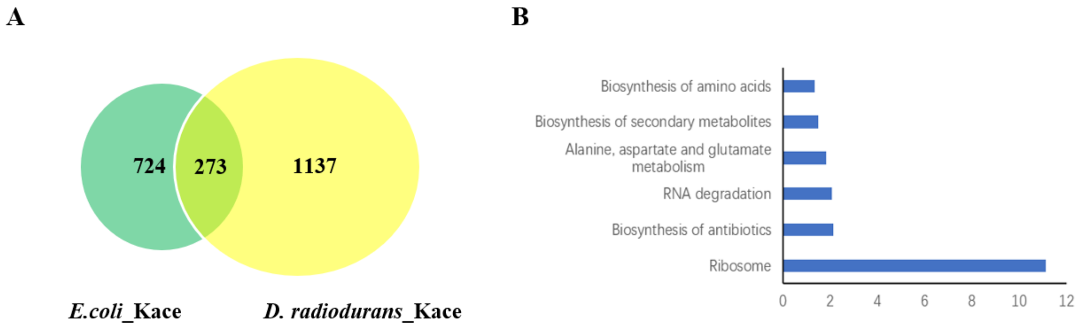

Fig.S2 Evolutionary conservation analysis of acetylated proteins between *D. radiodurans* and *E.coli*. (A) Overlap of acetylated proteins in the two kinds of bacterial. (B) KEGG pathways were enriched by DAVID corresponding to 273 shared acetylated proteins between *D. radiodurans* and *E.coli*.
